# Development and testing of an online community care platform for frail older adults in the Netherlands: a user-centred design

**DOI:** 10.1186/s12877-018-0774-7

**Published:** 2018-04-07

**Authors:** Sarah Willard, Ger Cremers, Yan Ping Man, Erik van Rossum, Marieke Spreeuwenberg, Luc de Witte

**Affiliations:** 1 Centre of Innovative Care and Technology (EIZT), Zuyd University of Applied Sciences, Henri Dunantstraat 2, 6419PB, Heerlen, The Netherlands; 2 School CAPHRI, Care and Public Health Research Institute (CAPHRI), Faculty of Health, Medicine and Life Sciences, Maastricht University, Maastricht, the Netherlands; 3 Centre for Assistive Technology and Connected Healthcare (CATCH), University of Sheffield, Sheffield, UK

**Keywords:** Ageing, frailty, Community dwelling, Care, ICT, Online community

## Abstract

**Background:**

Recent transitions in long-term care in the Netherlands have major consequences for community-dwelling older adults. A new paradigm expects them to manage and arrange their own care and support as much as possible. Technology can support this shift. A study has been conducted to explore the needs of community-dwelling frail older adults with regard to an online platform. An existing platform was subsequently modified, based upon these needs, resulting in an online community care platform (OCC-platform) comprising of care, health, and communication functions. The purpose of this platform was to support frail older adults in their independence and functioning, by stimulating self-care and providing reliable information, products and services.

**Methods:**

The study used a User-Centred Design. The development processes involved the following steps: *Step 1) Identification of the User Requirements.* To assess the user requirements, direct observations (*N* = 3) and interviews (*N* = 14) were performed. *Step 2) Modification of an Existing Online Platform.* Based upon *Step 1*, available online platforms were explored to determine whether an existing useful product was available. Two companies collaborated in modifying such a platform; *Step 3) Testing the Modified Platform.* A total of 73 older adults were invited to test a prototype of the OCC-platform during 6 months, which comprised of two phases: (1) a training phase; and (2) a testing phase.

**Results:**

An iterative process of modifications resulted in an interactive software concept on a Standard PC, containing 11 Functions. The Functions of ‘contacts’, ‘services’ and ‘messaging’, were by far, the most frequently used. The use was at its highest during the first 2 weeks of the testing and then its use steadily declined. The vast majority of the subjects (94%) were positive about the usability of the platform. Only a minority of the subjects (27%) indicated that the platform had added value for them.

**Conclusion:**

The overall prospect was that an OCC-platform can contribute to the social participation and the self-management competencies of frail older adults, together with their social cohesion in the community. In order to validate these prospects, further research is needed on the characteristics and the impact of online platforms.

## Background

Recent transitions in long-term care in the Netherlands have major consequences for community-dwelling older adults. They are now encouraged to stay at home for as long as possible. The responsibilities for care are now shifting from the health care system to older adults themselves, together with their social network. This shifting creates a new paradigm in which self-management competencies are highly valued. This new paradigm expects older adults to manage and arrange their own care and support, and if needed, to be supported by their social network (i.e. neighbours, family, friends) [1].

Most older people are relatively healthy, living independently in their own homes, and participating in their own community. Older adults who are vital and active will probably not experience many difficulties when self-managing their own care. Nevertheless, up to 38.5% of older adults are considered to be frail, with an increased risk for (further) functional decline and institutionalisation [2]. Frailty is defined as “a dynamic state which influences a person that experiences a loss in one or more domains of human functioning (physical, psychological, social)” [2]. This frailty can transpire due to a range of diseases and medical conditions [3]. The rapid increase of frail older adults with chronic diseases, multimorbidity, as well as complex health problems, affects the demand for health care services, while at the same time, financial resources and manpower are both shrinking [4, 5].

We will only succeed in this evolving transition when we search for comprehensive new and smart strategies, in a close collaboration between all of the stakeholders involved. Technology can support these strategies. An example is an online platform offering an infrastructure, in order to interlink people, provide access to professional services, as well as to promote (‘real life’) encounters and mutual activities [6–8]. An online platform is a coherent combination of hardware and software components that are able to provide a base upon which software applications can operate.

There are dozens of online health and welfare platforms. These mainly focus on delivering health care services and they are primarily developed from the perspective of health care professionals [9, 10]. Frail community-dwelling older adults especially need support in other domains, such as access to information, services, and communication [11].

We have conducted a study to explore the needs of community-dwelling frail older adults, with regard to an online platform. An existing platform was then modified, based upon these needs. This resulted in an online community care platform (OCC-platform) that comprised of health, well-being, and communication functions. The purpose of this platform was to support frail older adults in their independence and functioning, by stimulating self-care and providing reliable information, products and services. The main aims of the study were: 1) to modify an existing online platform, based upon the needs of frail older adults, and 2) to test this modified prototype platform on the usability and the feasibility of frail older adults.

## Methods

**Fig. 1:**
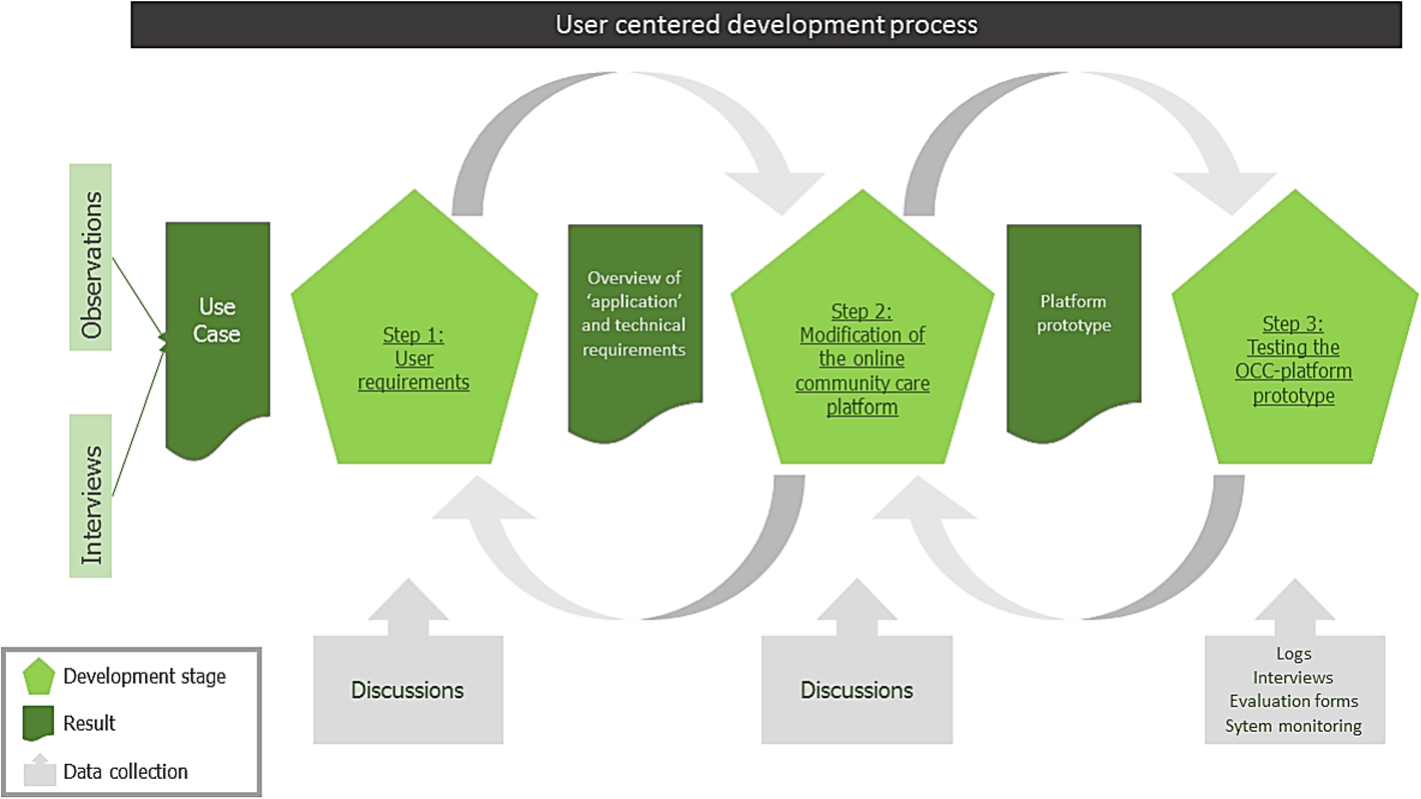
Overview of the steps in this study

We used a User Centred Design (UCD) [12]. A UCD is a framework in which the needs of the end users are given extensive consideration at each stage of the product’s design. The iterative nature of a UCD and the inclusion of various feedback loops are essential in adapting the technology to end user needs [11]. The user-centred development processes regarding the OCC-platform involved the following steps: 1) Identification of the User Requirements; 2) Modification of an Existing Online Platform; 3) Testing the Modified Platform. The study was carried out in Limburg, the most southerly province in the Netherlands. Figure 1 provides an overview of the steps in this study.

### Step 1: User requirements

In order to assess the personal needs and the requirements of frail older adults, direct observations and interviews were performed. Various organisations (e.g. a client-interest group, a hospital, and a local municipality) were asked to select suitable candidates (i.e. people who were living at home, and over 65, *N* = 17) to be included in this part of the study. This resulted in 17 frail older adults who participated in either the direct observations (*N* = 3), or the interviews (*N* = 14).

First, the direct observations were held during half a day and they were conducted at the homes of 3 older adults. The goal of the direct observations was to gain an insight into ‘an average day’ of older adults. The direct observations were non-invasive, i.e. aiming to minimally disturb their normal routines and behaviour due to the presence of the researchers. Afterwards, each participant was interviewed, in order to confirm the direct observations, by asking questions, such as: *“What problems do you encounter in daily life?”*; “*What problems do you encounter with respect to social contact?*”; *“How do you deal with these problems?*” The results from the direct observations were then analysed. From these analyses, corresponding topics were selected and they were then converted into the interview questions. The subsequent 14 interviews contained items such as social participation and communication. The interviews were carried out, in order to confirm the accuracy and the completeness of the results, arising from the direct observations. Examples of the topics and the interview questions are listed in Table 1.

**Table 1:** Interview topics and questions

Topics	Item	Examples of the Interview Questions
*Social Participation*	Social Contacts	With whom are you currently in touch with the most?
	Social Activities	In which (community) activities do you participate? What welfare services do you use?
	Care Contacts	With which health care professionals do you have contact?
*Communication Needs*	Remote Communication	What techniques would you like to use, in order to communicate with professionals or other people?
*Technology Needs*	Technology Features	If a device were to be developed, in order to enable remote communication, what requirements should it meet?
	Expectations Regarding Technology	How do you feel about remote communication?
	Affinity With Technology	Are you experienced when working with computers? For what purposes do you use a computer?

The results from the observations and the interviews were used to draft ‘use cases’, as well as narratives about the ‘modus operandi’ of the OCC-platform. This was all from the perspective of the proposed users [13]. These use cases were discussed and they were optimised by a geriatrician, a practitioner nurse, as well as the representatives of the frail older adults, together with four of the researchers. The final use cases were converted into user requirements, and then subsequently, they were divided into functional and technical requirements.

### Step 2: Development of the prototype platform

Based upon *Step 1,* the field of online platforms was explored, in order to determine whether an existing product was befittingly available. We sought for an existing useful online platform that was suitable for older adults, with a low cost, and available in the Dutch language. Furthermore, the owner of the platform had to be willing to modify it, based upon the user requirements. Consequently, several ICT companies were approached and consulted. Finally two companies were selected to work together and to develop a prototype platform. The company that was first selected presented the most promising online platform, as their software was of an ‘open source’, and thus, it was suitable for modification. Moreover, their platform, entitled “Cubigo”, contained certain features which had been labelled as being significant in the user requirements (i.e. communication and information on local services, events and activities). The choice was, therefore, to select their platform. Another company was selected to supply the ‘video call service’, since the platform of the ‘first’ company did not contain such a function.

The two companies modified the platform, based upon the user requirements that resulted from *Step 1*. Further optimisations were administered following discussions with the researchers and the representatives of the older adults. The proceedings were accomplished by arranging the following feedback-moments:

- Feedback-sessions regarding the layout: the researchers and the representatives of the frail older adults were asked to give their feedback on the layout of the platform (e.g. menu structure, visibility). The results were used to improve the platform’s layout.
- Tests by the researchers: the researchers were asked to test the prototype platform and to pay special attention to the usability of the platform and to any occurring inaccuracies. The results from these tests were used to improve and to optimise the usability of the platform. These processes were repeated several times.
- Tests by the representatives of the frail older adults: the prototype platform was tested in the home-environment of the representatives. The goal of these tests was to try-out several functions of the prototype platform (e.g. the video call services, text messages, and so on.). The representatives were also asked to pay special attention to the usability, the visibility, and the interactions. The results of these tests were used to improve and to optimise the online prototype platform.

After the abovementioned tests, the researchers, the local municipalities, and the care and welfare organisations, all provided content (i.e. information) for the platform. This resulted in a prototype platform that was ready for final testing.

### Step 3: Testing the OCC-prototype-platform

In order to test the prototype platform, a panel of participants was monitored for 6 months. Frail older adults were invited to participate via an information letter that was distributed by senior council workers, a hospital, care organisations, welfare organisations, in addition to general practitioners. Suitable participants (i.e. people who met any of the following criteria: aged 65 and older; at a risk of decline in physical, psychological, or social functioning; suffering from a geriatric syndrome; suffering from a specific somatic condition; living independently at home without a partner) were subsequently approached by the project members. The participants were introduced to the OCC-platform by way of a personal instruction and a demonstration. When the participants did not have access to a computer, or the Internet, these were made available to them free of charge. A total of 73 older adults were invited to participate in order to test the OCC-prototype-platform.

The testing comprised of two phases: (1) a training phase; and (2) a testing phase. Both of these phases consisted of tasks that had to be carried out by the participants individually, in their own home, and on their personal computer. The training phase, in order to become familiar with the platform, included various exercises in which the participants were asked to concentrate on specific functions. In the first week, the participants were asked to use the first three functions of the platform and to record their findings in a log. The participants were then contacted by telephone to discuss their findings and to receive a new assignment (i.e. to use the next three functions for a week and to record their findings). These procedures were repeated until the entire platform had been appraised. After these training exercises, the testing phase commenced. The participants were asked to continue using the platform freely and to log their experiences.

More participants were gradually included. The quantitative data of the user frequencies was automatically documented by the platform’s system, in log files, during the entire testing phase. Finally, all of the participants were interviewed in their own homes at the end of the testing phase. This interview consisted of 34 questions of which 23 addressed the usability of the OCC-platform (e.g. logging in to the platform, the menu’s structure and usability of the platform’s functions). The remaining 11 questions were dedicated to user experiences (e.g. did the OCC-platform contribute to feelings of safety and did older adults perceive the platform as having added value). The data were analysed by means of conventional content analyses [14].

### Data analyses

The interviews (*Step 1 and Step 3*) were transcribed and then they were qualitatively analysed. The data from the user’s log files was imported into SPSS and this was descriptively analysed (an overview of the frequency in which the tools were used and the trends of their use over time). Participants were gradually included into the testing phase (Step 3). As a result, the duration of participation varies from 15 to 30 weeks. To standardize the follow up period we restricted this to 15 weeks for every participant. We excluded the first three and last two weeks of data to avoid learning effects (i.e. we gave users the possibility to get accustomed to the platform) and fade-out effects. Therefore, we analysed the log files on the user frequencies restricted to 10 weeks for every participant.

## Results

### Wishes and needs regarding daily life, communication and technology

The goal of the direct observations was to gain an insight into the wishes and the needs of the frail older adults regarding their daily life and their communication. The direct observations were conducted at the homes of 3 frail older adults (2 females of 75 and 72 years and a male of 71 years), who all lived independently in their own home and were married. It turned out that they: (1) often stayed at home because it was perceived as being safer; (2) often received health care provided by informal and/or formal caregivers; (3) experienced limitations in their social contacts and they wished to have more face-to-face contact with their family and friends, or to be more involved in the community; (4) were dependent on transportation that was offered by their children or friends. Moreover, the 3 observed older adults felt that telecommunication technologies could improve their well-being and their feelings of safety. An important requirement, however, was that potential interventions should be user-friendly. For instance, the platform should have clear buttons or icons; there should be an easy menu structure; the platform should feature a short and understandable user manual; and it should be suitable for transport.

The interviews were conducted at the homes of 14 older adults and they were focused on their wishes and their needs regarding social participation, communication and technology. The participants (*N* = 14) had a mean age of 79 (SD = 6) and 9 of them were female. All participants lived independently in their own home and 12 of them were widowed. The results demonstrated that their social network consisted of children, siblings, neighbours and friends. Overall, the interviewees were unsatisfied with the degree of contact that they had with their children and siblings. Depending upon their health, they were involved in all kinds of activities (e.g. viewing sport, church attendance, and so forth.). The interviewees had contact with various health care professionals, such as visiting nurses, general practitioners, nurse practitioners, and physiotherapists. According to the interviewees, an OCC-platform should include the following functions: telephoning (including a video call service), text messaging, distress calls, and a calendar. Although 12 older adults were positive about the concept of an OCC-platform, the majority were not interested in screen-to-screen contact with health care professionals. Approximately 50% of the participants had a basic knowledge of computers and a little fewer than 50% owned a personal computer (PC). The PC owners generally used them for playing games, e-mailing, browsing online, administrative work, internet banking, and social media.

### Use cases

The results from the direct observations and the interviews were used to draft ‘use-cases’. Their structure was as follows: (1) case (e.g. general information, health problems, social problems, hospitalisation, and frailty); (2) intervention (e.g. family, an online platform offered them a support system); (3) ideal situation (e.g. frail people used the OCC-platform and they were satisfied with the functions/features and the usability).

### User requirements

Based upon the use cases, two lists of requirements were drafted by the researchers, namely (1) ‘Function Requirements’, and (2) ‘Technical Requirements’. Table 2 lists the required functions that are distributed over three topics: (1) General; (2) Community; and (3) Health.

**Table 2:** User requirements: functions

General	Community	Health
*Safety & Comfort* Personal Alarm; Smoke, Gas and Burglar Alarm; Intercom; Home Automation. *Services* Meals Delivery Service; Grocery Service; Banking Service; Job Services; Information Provided by Local Municipalities. *Communication* Video Calling Services; Chat Room; SMS. *News & Entertainment* Newspaper/Book (*Read Aloud*); Calendar with Audio and Reminders; Games to Play Individually.	*Social Safety* Distress Call; Good Morning Service provided by a Welfare Organisation. *Community* Service to arrange Transport; Information about Local Events and Activities; Interactive Games; Community Resources listed on an Interactive Map.	*Health Consultation* Primary Care Consultation (e.g. the practitioner nurse); Clinical Care Consultation (e.g. the clinical geriatrician). *Health Calendar* Medication Reminder; Appointment Reminder; Possibility to ask Questions Online for a Consultation; Results of the Consultation. *Health Advice* Lifestyle Advice/Counselling; Online Contact with Paramedics; Medication Counselling Online; Telemedicine (e.g. remote monitoring of psychical parameters).

The OCC-platform also had to meet several technical requirements: usability (the usage and the operation should be easy and intuitive, little to no affinity with computers needed); visibility (big screen for better visibility, use of icons, pictures, photos); flexible interaction (touch screen capability); accurate operation (robust operation, no technical failures during operation); convenience regarding transport (device should be portable, small, light, robust and wearable); it should be possible to add supplementary functions.

### The OCC-prototype-platform

**Fig. 2:**
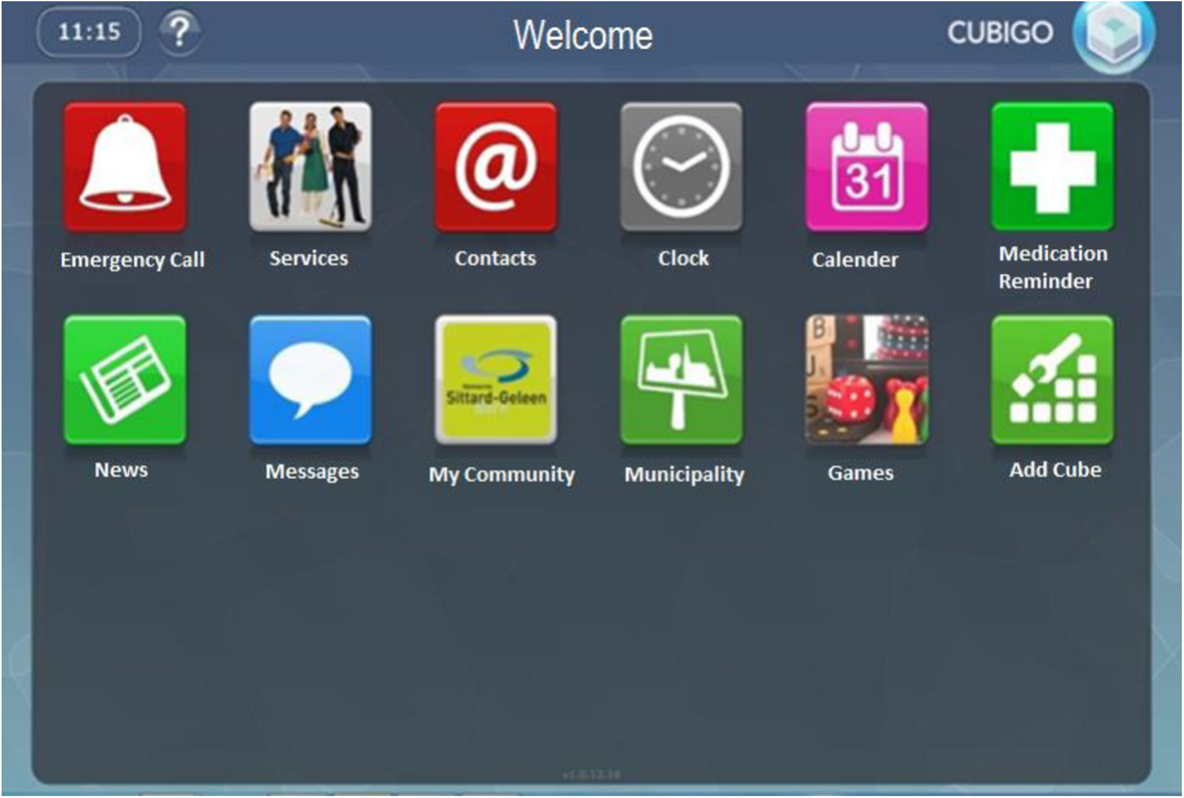
Screenshot of the OCC platform

An iterative process of modifications and optimisation resulted in an interactive software concept installed on a Standard PC, containing 11 Functions (cubes) in the Main Menu (Fig. 2): Emergency Call, Services, (Video) Contacts, Clock, Calendar, Medication Reminder, News, Sending and Receiving Messages, Information about the Community, Information from Municipalities, and Games.

Every function was comprised of submenus. The main menu also contained an extra function of “add cubes”, which gave the users the possibility of tailoring the basic screen according to their wishes, by adding, deleting and/or reordering functions (cubes). The development of the previously described OCC-prototype-platform resulted in considerably more time and energy than expected. In order to ensure a tangible involvement of health care providers, (i.e. health care providers who actually provided content for the OCC-platform), several visits and a lot of persuading was necessary. In the end, it took us half a year to persuade the health care providers to provide information content for the OCC-platform.

### Testing the OCC-prototype-platform

Of the 73 approached candidates, 55 older adults were included in the testing study. Eventually, 33 of them finished the entire monitoring period of 6 months. Eventually 22 participants withdrew from the study due to hospitalisation, or because they found participation in the study too time consuming. Of the 33 participants that completed the study, 20% were younger than 70 (the youngest was 65) and 60% were 70–80 years old. Eight participants did not have a computer or an Internet connection. These were made available to them, free of charge.

### User frequencies

**Fig. 3:**
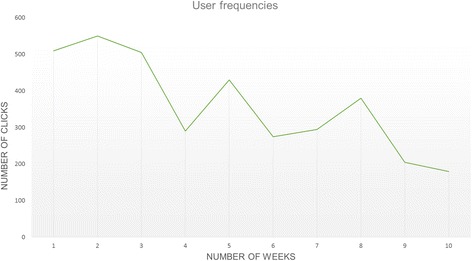
Frequency of use of the OCC-platform distributed over 10 Weeks

As portrayed in Fig. 3, the frequency of use of the OCC-platform generally decreased over time, with a few peaks between weeks 4 and 5 and weeks 7 and 8. In the first 2 weeks of the study, the participants clicked 500–550 times (all of the functions included). This frequency decreased to approximately 170–205 clicks per week over the last 2 weeks. These data relate to 33 participants with complete data over the 10 week period.

**Fig. 4:**
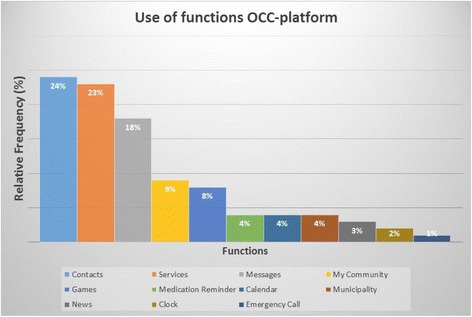
User frequencies per platform function

Figure 4 further visualises the frequency of use per function. It shows that the functions of ‘contacts’, ‘services’, ‘messages’ and ‘my community’ were the most frequently used (74% of total usage). Conversely, the functions of ‘news’, ‘clock’ and ‘emergency call’ were rarely accessed (6% of total usage).

### Usability

The main topics regarding the usability of the platform were: logging into the platform, the design of the main menu and submenus, the format and structure of the main menu and sub menus, the visibility and recognisability of the buttons, the readability of the text, the brightness of the colours, as well as the clarity and the audibility of the sounds. In general, 33 participants gave a positive score for the usability of the OCC-platform (94% stated that they found the platform easy to use). Nevertheless, several recommendations were made. The most important of these was that the OCC-platform login should be made easier (username and password were often forgotten) and that the number of functions and the arrangement of the functions in the main menu should be individually adaptable. Additionally, the functions and the sub functions gave too much information (too many choices for the selections, too many actions required).

### User experiences

The comments of the participants with respect to their experiences with the platform vastly diverged. The majority of the participants (73%) disagreed with the proposition: "the platform adds value to my daily life". Possibly they perceived themselves as being vital and active and not (yet) in need of support by the OCC-platform. Only 9 of the 33 subjects (27%) indicated that "the platform provided feelings of safety". Two thirds (67%) of the older adults preferred communicating via video, over communication by telephone. Furthermore, 18 of the 33 (55%) had the intention of using the platform actively after the testing period and 27 of the 33 (82%) reported that they would recommend the platform to others. A few of the participants advised the researchers to include some additional functions, such as: home automation (opening and closing the curtains and doors, controlling the heating); monitoring bodily functions (heart rate, blood pressure); a tool for matching care demanders and volunteers; hobbies; public transportation; taxi transportation; and games.

## Discussion

Based upon the user requirements of the frail older adults, an online community care platform was developed and tested on its usability and its feasibility over a six months period. The main aims of the study were: 1) to modify an existing online platform based upon the needs of the frail older adults, and 2) to test this modified prototype platform on its usability and its feasibility among frail older adults.

The most pertinent needs of the participating frail older adults were that they experienced limitations in their social contacts and they wished to have more face-to-face contact with their family and friends, or to be more involved in the community. Overall, the participants felt that telecommunication technologies could improve their well-being and their feelings of safety (i.e. the frail older adults seemed to be receptive to the idea of an OCC-platform). The OCC-platform was modified and optimised by two companies based upon the user requirements. This process resulted in an accessible interactive software concept on a Standard PC, containing 11 Functions (cubes) in the Main Menu. The OCC-platform functions of ‘contacts’, ‘services’ and ‘messaging’ were by far, the most frequently used. The usage of the platform was at its highest during the first 2 weeks of the testing study and then it steadily declined. Although the vast majority of the subjects were positive about the usability of the platform, only a minority (27%) indicated that the platform had added value.

An explanation for the decline in user frequencies was that the first part of the testing study, the training phase, was guided intensively by the researchers. In the second part, the platform was used ‘freely’ without external incentives to do so. The temporary increase of activity in week 7–8 was probably caused by an announcement that in those weeks, there would be an evaluation of activities by the researchers. These results have indicated that the OCC-platform did not produce sufficient incentives to keep the users active. In previous research, a similar low usage of online, or eHealth interventions, has been reported [15–18]. For example, the Dutch eHealth Monitor 2016 demonstrated that despite the availability of many eHealth tools, the end users hardly adopted them. Krijgsman et al. explained that the scarce adaptation of eHealth resulted from a ‘lack of awareness’ (i.e. the end users were unaware of the potential, the possibilities, or even the existence of eHealth). Furthermore, the end users would often rather stick to old habits, than adopt themselves to new ways and means (e.g. using the telephone to make appointments, instead of an online tool, filling out forms on paper, instead of online, and so forth). In order to encourage the adaptation of eHealth, it would be important to actively stimulate the usage and to promote eHealth [18]. Additionally, it would be vital that an eHealth OCC-platform contained content and functions that were of a value for its users.

Furthermore, as explained regarding the OCC-platform-prototype, it took us half a year to persuade the health care providers to provide information for the OCC-platform. It seemed that they partly did not have a clear idea yet of their role or the platform’s added value. Also, it is conceivable that some of them dreaded a possible increase in workload (e.g. using e-consults or video-calling tools, to register and share information via a platform etc.).

As the results have shown, the functions of communication (e.g. messaging) and participation (e.g. my community) were often used. Moreover, the participants seemed to have a preference for communicating via video call services, instead of communicating by telephone. Makai and colleagues [15] also reported that communication functions (e.g. messaging) were the preferred functions by older users. These results have indicated that an OCC-platform would have the potential to support community-dwelling frail older adults, especially in communication. However, this would only be true for communication between the end users and their family or friends. This would not apply to communication with the health care providers.

Only a quarter of the subjects indicated that the platform had added value. A possible explanation for this might be that OCC-platform technology still needs to be improved (e.g. an easier login), that it lacks specific relevant functions (e.g. transport or home automation), or that the information content was not up to date.

### Practical recommendations

The results have suggested that an OCC-platform has the potential to support community-dwelling older adults, provided that the platform contains content and functions that are of value for its users, in addition to the fact that end users are informed about the platform. In order to accomplish this, the study has several practical recommendations. Firstly, give the end users more control and ownership of the platform. For instance, when the platform is implemented on a community level (e.g. a neighbourhood), it is recommended that local residents are involved, when the customisation of the OCC-platform takes place. Secondly, the membership of the local health care providers and the local (elderly) associations should be involved, in order to make arrangements about their role in providing information content. Thirdly, there is a need to actively stimulate the usage and to promote the OCC-platform via the aforementioned parties. Thus, by intensifying the involvement of the end users, the health care providers, together with the local associations, we can safely assume that the OCC-platform will be promoted. It would then have more added value, as well as containing appealing and up to date content.

The fourth recommendation concerns the manner in which the OCC-platform is to be financed. At present, most of the funding is collected from local entrepreneurs (e.g. bakery, barber shop) who display and advertise their services on the OCC-platform. Due to these business cases, the continuity of the platform would entirely be dependent upon whether entrepreneurs want to use this platform as a promotional medium. Entrepreneurs, however, are not inclined to pay licence fees, in order to advertise on a promotional medium, from which they will only find a few customers. Conversely, the few customers (users) who actually use the OCC-platform can possibly be explained by a lack of the entrepreneurs to display their services. The business case of an OCC-platform will only work if *all parties* become involved (e.g. health care providers) and are willing to invest financially in an OCC-platform.

### Limitations and strengths of the study

This study has some limitations. In view of the modest sample size (especially in the direct observation and the needs part), the results should be interpreted with some caution. For instance, the results regarding the user requirements may not necessarily be applicable to all or other groups of older adults. The strength of this study was that the needs of the target group for this innovation (i.e. the frail older adults) were given extensive consideration at the selection, modification, and the testing of the OCC-platform. Another advantage was that the OCC-platform was tested in the home environments of the frail older adults.

## Conclusions

The overall prospect is that an OCC-platform can contribute to the social participation and the self-management competencies of frail older adults, as well as with their social cohesion in the community. In order to validate these prospects, further research is needed on the characteristics and the impact of online platforms [17].
